# Does the diameter of the thoracic inlet influence the formation of retrosternal goiter?^☆^^☆☆^

**DOI:** 10.1016/j.bjorl.2025.101597

**Published:** 2025-05-29

**Authors:** Leonardo Daniel Manzano Pasquel, Daniel Abreu Rocha, Yasmin Laryssa Moura Guimaraes, Gustavo Fernandes de Alvarenga, Mauricio Kase, Júlia Scomparin Magalhãnes, Regina Lúcia Elia Gomes, Ledo Mazzei Massoni Neto, Renata Lorencetti Mahmoud, Leandro Luongo de Matos, Vergilius José Furtado de Araujo Filho, Claudio Roberto Cernea

**Affiliations:** a Universidade de São Paulo (USP), Faculdade de Medicina (FM), Departamento da Cirurgia de Cabeça e Pescoço, São Paulo, SP, Brazil; b Universidade de São Paulo (USP), Faculdade de Medicina (FM), Departamento de Radiologia, São Paulo, SP, Brazil

**Keywords:** Goiter, Retrosternal goiter, Thoracic approach, Thoracic inlet

## Abstract

**Objective:**

Find an association between diameter of the Thoracic Inlet (TI) and RSG to determine whether the TI would function as a natural anatomical barrier to prevent the passage of the goiters to the thoracic cavity.

**Methods:**

A retrospective study was conducted with patients submitted to total thyroidectomy, with goiter greater than 50 cm^3^, who underwent Computed Tomography (CT) preoperatively to measure the TI volume. The values obtained from each continuous variable of parametric distribution were organized and described as mean and standard deviation. The distributions were defined as non-parametric by the Kolmogorov-Smirnov test. The Mann-Whitney test was used to compare two sample populations.

**Results:**

A total of 173 patients submitted to total thyroidectomy were evaluated, and 54 patients met the inclusion criteria of the study. 85.2% were female, with a mean age of 57-years. The mean diameter of the TI was 5679 mm^2^. 42% of the patients presented some degree of RSG. The distance below the TI ranged from 0.2 to 5 cm.

**Conclusion:**

No significant association was found between diameter of the thoracic inlet and retrosternal goiter, demonstrating that RSG can be present regardless of the TI diameter. Statistically significant association was observed between patients with larger thyroid volume and the likelihood of this tissue to extend to the thoracic cavity.

**Level of evidence:**

Level IV.

## Highlights

• A total of 173 patients submitted to total thyroidectomy for substernal goiter mean diameter of the TI was 5679 mm^2^.
• The distance below the TI ranged from 0.2 to 5 cm.
• No significant association was found between diameter of the thoracic inlet and retrosternal goiter, demonstrating that RSG can be present regardless of the TI diameter.
• Statistically significant association was observed between patients with larger thyroid volume and the likelihood of this tissue to extend to the thoracic cavity.

## Introduction

**Fig. 1:**
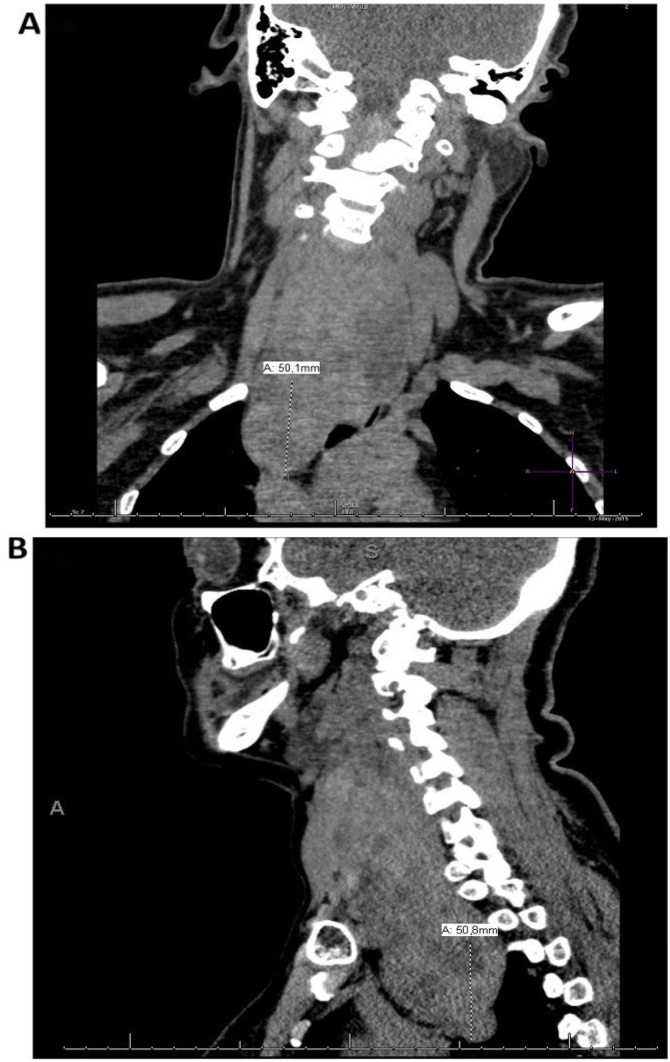
Patient with 5 cm retrosternal goiter below the thoracic inlet (total volume 83 cm^3^). (A) Coronal plane, (B) Sagittal plane.

Retrosternal (or substernal or intrathoracic) Goiter (RSG) was first described by Haller in 1749, and it is defined as growth or spread of the thyroid gland below the plane of the Thoracic Inlet (TI).^1^ The literature classifies RSG as one that presents more than 50% of its mass lying inferior to the thoracic inlet (Fig. 1). It is estimated that <5% of the world’s population has goiter and that RSG is present in 2%‒26% of all thyroidectomies, depending on the defining criteria. The reason why it is not possible to accurately estimate the incidence of RSG may be the different definitions used for both management and classification of this condition. Therefore, there are no standards for communicating findings and results among physicians, or for assisting with preoperative planning.^2^, ^3^

Embryology of the thyroid gland must be remembered when referring to its location. The gland develops from an endodermal thickening located on the floor of the pharynx (which will later form the foramen cecum) before descending to its final position in the neck. Most commonly the thyroid loses connection with its origin, however, it sometimes maintains a certain connection with the middle and posterior thirds of the tongue (via the thyroglossal duct) and with the pyramidal lobe in the isthmus.^4^, ^5^ This type of thyroid development explains the genesis of the thyroglossal duct and the presence of the gland in ectopic positions, such as the rare conditions of lingual thyroid and primary intrathoracic goiter.

Thoracic inlet, or superior thoracic aperture, refers to the opening at the top of the thoracic cavity, and the dimensions of this space are roughly 5 cm anteriorly and 10 cm transversely. The TI is bounded by the upper border of the manubrium anteriorly, the first Thoracic vertebra (T1) posteriorly, and the first rib and costal cartilage laterally.^6^ However, there is no uniformity among clinically oriented anatomy books on TI because, according to Snell, the thoracic cavity communicates with the base of the neck through the thoracic outlet.^7^, ^8^

Ultrasound Scan (US) is considered the preferred method for acquiring thyroid images, and it can accurately measure its volume. It enables simple, easy access without exposure to radiation, but also presents certain method-related limitations (access to intrathoracic extension assessment) and limitations regarding operator dependence.^9^ Computed Tomography (CT) is more accurate for assessing RSG, with precise identification of lesion limits and their relationship with the intrathoracic structures. According to the studies by Michel and Bradpiece, for the diagnosis of RSG, CT, thyroid scintigraphy and chest X-Ray showed sensitivity levels of 100%, 77% and 59%, respectively.^3^, ^9^

The literature does not explain why some goiters have an exclusively cervical component and some have an intrathoracic component. Due to the close relationship between the TI and the cervical region and the thorax, it is considered whether there is an anatomical relationship between the formation of goiter and its retrosternal component.

## Methods

### Patients and treatment

This study evaluated 173 patients undergoing total thyroidectomy at the Head and Neck (H&N) Surgery Center of the Hospital das Clínicas, College of Medicine, University of São Paulo (HC FM-USP) between February 2017 and December 2018. Demographic data, type of surgical procedure, imaging exams, and type of goiter (cervical or intrathoracic) were obtained from the patient’s medical records. Inclusion criteria for the study were as follows: thyroid volume >50 mm^3^ on preoperative US and CT.

### Ultrasound scan

The staff of the Radiology Institute (INRAD) of the HC FM-USP performed US using 9‒12 MHz high frequency linear probes. These scans were carried out in a variety of devices. Thyroid volume was calculated using a simple method described by Brunn et al., in which the volume of each lobe, considered as an ellipsoid structure, is determined by multiplying the Anteroposterior (AP), Transverse (T) and Longitudinal (L) diameters by the volume correction factor 0.5239. In addition to the total thyroid volume, the volume of the dominant thyroid lobe was also recorded.

### Computed tomography

Each patient underwent a chest CT scan on different 16, 64 or 325 channel scanners. Multislice CT scans were performed according to local routines. The patients were examined supine with the neck in neutral position, with arms alongside the body, with or without intravenous contrast enhancement, and 1‒5 mm slice thickness was acquired and reconstructed in the axial, coronal and sagittal planes.

**Fig. 2:**
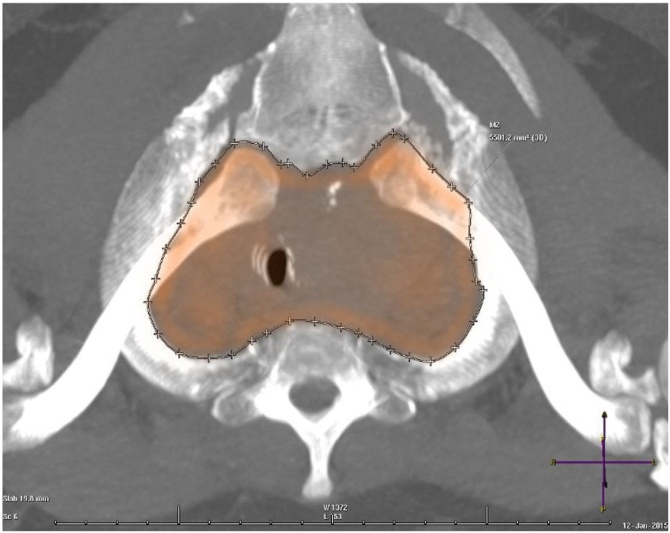
Thoracic Inlet (TI) measurement.

We collected and revised images from the institution’s health records to obtain relevant patient information. Imaging studies were initially reviewed to determine patient eligibility. Substernal extension was defined as any part of the thyroid gland extending beyond the sternal notch with the patient in supine position, as detected by CT. In order to measure the TI, Multiplanar Reconstructed (MPR) images from CT studies of the neck of the selected patients were used. Angulation was performed with reference to the axial plane of the first costal arches, using MIP (Maximum Intensity Projection) reconstruction as needed. The lateral limits were the superior and internal borders of the first costal arches, the posterior limit to the anterior border of the vertebral body of D1, and the anterior limit to the posterior border of the sternal manubrium. Measurements were given in mm^2^ (Fig. 2).

### Statistical analysis

The collected data were entered into a Microsoft Excel spreadsheet and processed using the SPSS© 24.0 statistical software (SPSS© INC; Illinois, USA). The values obtained by studying each continuous variable of parametric distribution were organized and described as mean and standard deviation. Absolute and relative frequencies were used for the categorized variables. The distributions were defined as non-parametric by the Kolmogorov-Smirnov test. The Mann–Whitney test was applied to compare two sample populations.

## Results

This study evaluated 173 patients with compressive symptoms and goiter >50 cm^3^ in total volume determined by US. All patients underwent total thyroidectomy at the H&N Surgery Department at the HC-FM-USP. Fifty-four patients with preoperative CT were included in the study, 46 (85.2%) of them were female with a mean age of 57-years (Table 1).

**Table 1:** General descriptive analysis.

		Number of cases	Percentage
Sex	Female	46	85.2%
	Male	8	14.8%
Presence of RSG	Cervical	31	57%
	Intrathoracic	23	42%
Nodules evidenced in US	Without nodules	2	3.7%
	Unilateral	9	16.7%
	Bilateral	43	79.6%

RSG, Retrosternal Goiter; US, Ultrasound.

Imaging techniques were used to evaluate inclusion criteria and measurement values of the study patients. Using preoperative US, the minimum and maximum values of the operated goiters were determined: 50.2 and 433 cm^3^, respectively. It was verified that 43 (79.6%) of the patients had bilateral nodules, 9 (16.7%) presented simple nodules, and 2 (3.7%) did not have thyroid nodules.

The TI diameters of the 54 patients evaluated were obtained through the aforementioned calculation, namely, mean diameter = 5679 mm^2^, minimum diameter = 4524 mm^2^, and maximum diameter = 7359 mm^2^.

Presence of thyroid tissue below the TI was considered as RSG, and some grade of it was observed in 42% of the patients. The distance from the TI to the end of the lower thyroid pole ranged from 0.2 to 5 cm. The right lobe was the most commonly found at the substernal level (52% of RSG), and only 9% of RSG were bilateral. Males were the least affected, with only four of the 23 patients with the disease. Nodules were present in all thyroids, and 83% of the patients presented bilateral nodules (Table 2).

**Table 2:** General descriptive analysis.

		Age at the time of surgery	Preoperative US ‒ Gland size	Thoracic inlet size at CT^a^	Thyroid tissue below the TI^b^
Mean		57.12	119.4	5679.28	1.6
Minimum		4	50.2	4524	0.2
Maximum		79	433.0	7359	5.0
Percentile	25	48.40	63.4	5366.75	0.8
	50	59.83	92.4	5660.50	1.3
	75	70.23	133.9	5939.25	2.0

US, Ultrasound; CT, Computed Tomography; TI, Thoracic Inlet.

a Size in mm^2^.

b Tissue measured in cm.

Of all the patients assessed, 31 presented no grade of RSG; 87% of the cases were female and 24 had bilateral nodules. The mean volume of thyroid gland removed was 97 cm^3^, with a maximum value of 347 cm^3^ (Table 3).

**Table 3:** Comparative data of non-diving goiter and diving goiter.

		Age at the time of surgery	Preoperative US ‒ Gland size	Thoracic inlet size on at CT^a^	Thyroid tissue below the TI^b^
Retrosternal goiter	Cases	23	23	23	23
	Mean	57.38	149.5	5664.00	1.648
	Minimum	18	51.1	4524	0.2
	Maximum	79	433.0	7237	5.0
Goiter	Cases	31	31	31	0
	Mean	56.93	97.042	5690.61	
	Minimum	4	50.2	4628	
	Maximum	77	347.6	7359	

US, Ultrasound; CT, Computed Tomography; TI, Thoracic Inlet.

a Size in mm^2^.

b Tissue measured in cm.

Evaluation of goiter by US showed larger volumes in patients with RSG (p = 0.011; Mann-Whitney *U* test), but no difference was observed with respect to laterality. Regarding TI, no statistically significant difference was observed between the area and presence of the RSG component (p = 0.733; Mann–Whitney *U* test). Table 4 shows all these analyses.

**Table 4:** Comparison between goiter volume at Ultrasound (US) and Thoracic Inlet (TI) area at Computed Tomography (CT) and Retrosternal Goiter (RSG).

Variable	RSG^a^	Goiter^a^	p-value^b^
Gland volume at US (cm^3^)	97.0 ± 57.9	149.5 ± 96.8	0.011
Right lobe volume at US (cm^3^)	41.3 ± 34.9	54.2 ± 45.3	0.310
Left lobe volume at US (cm^3^)	49.6 ± 43.4	83.7 ± 94.9	0.162
Volume of the isthmus at US (cm^3^)	7.3 ± 10.7	11.3 ± 17.2	0.711
TI area (mm^2^)	5.690.6 ± 632.3	5.664.0 ± 539.3	0.733

a Mean ± Standard Deviation.

b Mann–Whitney *U* test.

## Discussion

Among the different definitions of goiter found in the literature, the most commonly used defines it as thyroid gland larger than twice the normal volume.^10^ When the lower pole of the thyroid can be located on palpation during physical examination, there are increased chances of occurrence of RSG, and this usually occurs in goiters >50 cm^3^, with need for several supportive imaging exams. There is not a single classification of RSG,^11^, ^12^ thus there is a very wide variation in its incidence in the literature;^2^, ^3^, ^10^ a higher incidence (42%) was found in the present study because RSG was categorized as any thyroid tissue below the TI.

Retrosternal goiter is pathology of exclusive surgical resolution, and it can be safely extracted mainly through a cervical incision. The use of routine CT to assess patients with goiter is uncommon, and the decision to perform this test will depend on several other symptoms or signs. As surgeons, we agree that CT assists with determining potential complications, thoracic gland size, possible difficulties for anesthesia, and the possibility of an extra-cervical procedure to approach RSG.^11^, ^12^, ^13^ Several authors have suggested that goiter extension to the level of the aortic arch, especially when associated with tracheal involvement or displacement of a larger vessel, increases the probability of a sternotomy.^1^, ^3^, ^10^

## Conclusion

There are no studies in the literature reporting the existence of any degree of influence of the Thoracic Inlet (TI) diameter and the presence of Retrosternal Goiter (RSG), thus we proposed to correlate these factors in our patients. Our results showed that this association is not significant, demonstrating that RSG may be present regardless of TI size. Statistically significant association was observed between patients with larger thyroid volume and the likelihood of this tissue to extend to the thoracic cavity.

## Declaration of competing interest

The authors declare no conflicts of interest.
